# Development of Brazil Nut Oil‐Based Oleogel and Emulsion Gel: A Comparative Study of Structural and Physicochemical Properties

**DOI:** 10.1111/1750-3841.71401

**Published:** 2026-08-25

**Authors:** Rogério Willian Silva dos Santos, Md. Jannatul Ferdaus, Marcos Rogério Mafra, Roberta Claro da Silva, Tirzhá Lins Porto Dantas

**Affiliations:** ^1^ Department of Chemical Engineering Federal University of Paraná (UFPR), Polytechnic Center Curitiba Brazil; ^2^ Food and Nutritional Sciences Program, Department of Family and Consumer Sciences North Carolina Agricultural and Technical State University Greensboro North Carolina USA

**Keywords:** *Bertholletia excelsa*, carnauba wax, gelatin, healthy fat, structured lipid

## Abstract

In this study, Brazil nut oil (BNO) was structured either as oleogels (OG) using carnauba wax (CW) or as emulsion gels (EG) using gelatin at water:oil ratios of 1:1, producing systems intended as models for solid fats. The effects of structuring methods and different gelling agent concentrations were comparatively evaluated in terms of their structural, physicochemical, and thermal properties. Visual analysis demonstrated that BNO can be effectively structured with 6% CW, while 2% gelatin was sufficient to induce the transition from liquid oil to a semi‐solid material. Polarized light microscopy (PLM) revealed distinct structural organizations contributing to their respective structural stability, with crystalline structures in OG and emulsion‐filled networks in EG. Regarding oil binding capacity (OBC), it increased significantly with higher CW concentrations, rising from 30.63% ± 1.34% (BOG2) to 83.65% ± 1.90% (BOG10) (*p* < 0.05). Between EG samples, all OBC results were statistically similar, reaching approximately 100% oil retention (*p* < 0.05). DSC analysis revealed not only a higher melting onset but also greater enthalpy for BEG (491.5–726.8 J/g) compared to BOG (1.5–17.8 J/g), demonstrating superior thermal stability and network organization of gelatin‐based gels. Furthermore, rheological tests showed that BEGs exhibited a stable linear viscoelastic region (LVR) (G′ > G″ up to >12% strain) and a higher crossover point (∼100%), indicating greater structural strength than BOGs. These findings provide a foundation for future studies exploring their potential use as models for solid fats.

## 1 Introduction

Although solid fats contribute significantly to food structure and sensory properties (Zampouni et al. 2024), their consumption has been strongly linked to various health risks, particularly cardiovascular diseases (Guo et al. 2023; Frías et al. 2023; Langley et al. 2020; López‐Pedrouso et al. 2021). Consequently, there are efforts to replace trans and saturated fats with healthy oils (Ferdaus et al. 2024; Mahmud et al. 2024; Blount et al. 2025; Silva et al. 2023; Oyom et al. 2024). For instance, the World Health Organization (WHO) has issued guidelines on saturated and trans‐fatty acid intake, which state that saturated fat intake should be less than 10% of total daily energy consumption and replaced with polyunsaturated fatty acids (PUFAs) and monounsaturated fatty acids (MUFAs) to minimize the incidence of cardiovascular diseases (WHO 2023).

However, the simple substitution of saturated fats with unsaturated oils poses several challenges for the food industry, as solid fats contribute to key physicochemical properties of food products (Gao et al. 2024; Q. Wang et al. 2024). In response, fat mimetics have emerged as promising alternatives to conventional fats (Ferdaus et al. 2022; Silva et al. 2023). These substitutes are created from proteins, healthy oils, or polysaccharides that aim to replicate the functional properties of solid fats while minimizing health risks (Vélez‐Erazo et al. 2022; Cen et al. 2024; Huang et al. 2023; Xie et al. 2023).

In this context, an approach known as organogelation has been developed using conventional oils to produce oleogel (OG) as a healthy substitute for solid fats (Guo et al. 2023; Manzoor et al. 2022). This concept is based on a high amount of oil entrapped within a three‐dimensional network using one or more organogelators (10% maximum amount), also called gelling or structuring agents (Z. Huang et al. 2023). Carnauba wax (CW) has been used as a gelling agent due to its high melting point (81°C–86°C), low solubility, and relative inertness and stability, as stated by the European Food Safety Authority (Susmita Devi et al. 2022). Compared to other waxes, CW has been mentioned as ideal for applications requiring high structural integrity, such as fat replacers in high‐temperature food processing (Alshehri et al. 2025).

Emulsion gels (EGs) also have emerged as an alternative strategy for replacing solid fats with healthier vegetable oils, offering improved stability, safety, solubilized hydrophobic and hydrophilic components, and nutritional benefits compared to the OGs (Lin, Kelly, and Miao 2020; Farjami and Madadlou 2019; Wang et al. 2025). EGs are structured systems defined by gel‐like networks and solid‐like textural properties (Keramat and Golmakani 2024), formed through the incorporation of gelling agents into various types of emulsions (O/W, W/O, W/O/W, or O/W/O). These agents promote the formation of a three‐dimensional matrix that enhances the stability of the dispersed phase (Czapalay and Marangoni 2024). For this purpose, gelatin, a non‐globular, natural amphiphilic protein derived from collagen, exhibits excellent gelling, emulsifying, and film‐forming capabilities (Yan et al. 2023). Due to its characteristics of gelation, biocompatibility, wide source, low cost, and nontoxicity, gelatin is considered a promising raw material for EGs application (Mao et al. 2022).

These innovations present an opportunity to utilize healthy vegetable oils that remain underexplored in industrial applications, particularly Amazon oils. Among the 84 oilseed species identified in the region by Pesce (2009), *Bertholletia excelsa* oil (Brazil nut oil, BNO) stands out from conventional oils due to its high MUFAs content, antioxidant activity, and unique selenium concentration, which contribute to cardiovascular and anti‐inflammatory benefits (Yang 2009; Cominetti et al. 2012; Vasquez et al. 2021; Cardoso et al. 2016). These advantages position BNO as a promising alternative for designing structured lipids, while simultaneously fostering sustainable development through the valorization of local biodiversity, support of rural livelihoods, and mitigation of environmental impacts compared with conventional oil crops.

The objective of this study was to develop novel OGs and EGs using BNO as the continuous phase and CW and gelatin as gelling agents, respectively. Specifically, the study evaluated the effects of varying concentrations of gelling agents and processing methods on structural, physicochemical, and thermal properties of OGs and EGs. Although OG and EG systems have been widely investigated as fat models, studies directly comparing these two structuring approaches using the same oil matrix remain limited. In this context, the present work provides a systematic and side‐by‐side evaluation of BNO structured as both OG and EG, enabling a direct assessment of how different gelation mechanisms influence their properties. This approach offers new insights into the relationship between structure and functionality in lipid‐based systems and supports the potential development of tailored fat substitutes based on specific application requirements. Besides, the findings contribute to a better understanding of structured lipid systems and may support future studies exploring the use of Amazon oils as alternative lipid materials, thereby contributing to the development of regional bioeconomy.

## 2 Methodology

### 2.1 Materials

CW (melting point of 81°C–86°C) was purchased from Fisher Scientific (Hampton, NH, USA), unflavored and colorless food grade gelatin (Type A) from MP Biomedicals (Santa Ana, CA, USA), Tween 20 from Sigma‐Aldrich (St. Louis, MO, USA). Additionally, deionized water was used in all experiments.

### 2.2 Methods

#### 2.2.1 Extraction of Brazil Nuts Oils (BNO)

The BNO was extracted as described by our research group using n‐hexane (Neon, 99.8% purity) as a solvent (Santos et al. 2025). Thus, Ultra Turrax‐assisted extraction (UTAE) was conducted using a T25 digital Ultra Turrax (IKA‐Werke, Staufen, Germany) under the following process conditions: a time of 27 min and a solvent‐to‐solid ratio of 26 mL/g. The oils were stored in amber glass and kept at −5°C until the development of Brazil Nuts oil oleogels (BOG) and Brazil Nuts oil emulgels (BEG). The BNO fatty acid composition was analyzed in our previous research, finding it comprises of 28.53% of saturated fatty acid, primarily palmitic acid (C16:0) at 15.68%, stearic acid (C18:0) at 12.85% and 71.45% of unsaturated fatty acids, including oleic acid (C18:1) at 37.56% and linoleic acid (C18:2) at 33.89% (Santos et al. 2025).

#### 2.2.2 Preparation of Brazil Nuts Oil Oleogels (BOG)

The BOGs were prepared according to the direct method based on the methodologies of Airoldi et al. (2022) and Moghtadaei et al. (2018), with adaptations. Thus, 15 g of the oil (solvent) was heated to 100°C under magnetic stirring (350 rpm). Once the oil temperature was reached, CW, as the gelling agent, was slowly added (2, 4, 6, 8, and 10% w/w) and mixed until dissolution (5 min). After the complete incorporation of CW, the samples (in liquid form) were transferred to the test tubes. Then, the samples were kept in a static condition at room temperature (20°C ± 4°C) for 1 h to structure and stabilize. Finally, the samples were stored at 4°C until characterization. The samples were coded as BOG2, BOG4, BOG6, BOG8, and BOG10 regarding the CW concentration.

#### 2.2.3 Preparation of Brazil Nuts Oil Emulgels (BEG)

The EGs were prepared by dissolving % Tween 20 (a sorbitan monolaurate) in ultrapure water (v/v) at 25°C. In the second step, gelatin was added in the following proportions: 2%, 4%, 6%, and 8% (m/v), and the mixture were left to wait for 30 min to complete the hydration. After hydration, BNO (solvent) was added to the gelatin solution at a 1:1 (v/v) ratio and homogenized using a high‐speed disperser (digital Ultra‐Turrax, T25, IKA, Germany) at 10,000 rpm for 2 min. Then, all samples were dehydrated at 50°C for 24 h or until they reached approximately 25% moisture content (MA37‐1US Infrared Moisture Analyzer, Sartorius). After drying, the samples were homogenized again using the Ultra‐Turrax under the same conditions described previously (10,000 rpm for 2 min). Regarding the moisture level, it was selected based on preliminary tests, in which the emulsion gels exhibited gel‐like behavior and structural stability specifically around this value. Consequently, after complete dispersion, they were transferred to test tubes and maintained in a static condition at room temperature (20°C ± 4°C) for 1 h to facilitate structuring and stabilization. Finally, the samples were stored at 4°C and then characterized. The samples were coded as BEG2, BEG4, BEG6, and BEG8 regarding the gelatin proportion.

### 2.3 Characterization

#### 2.3.1 Macroscopic Appearance Analysis

The macroscopic appearance analysis was developed according to R. Wang et al. (2024), with modifications. Thus, 5 g of the sample was poured into a test tube, cooled to 25°C, and then stored in a refrigerator at 4°C for 24 h. After 24 h, the samples were then placed at 25°C for 2 h, and the test tubes were inverted to observe the fluidity of the samples and determine whether they had gelified or not.

#### 2.3.2 Polarized Light Microscopy (PLM)

BOGs and BEGs microstructure were analyzed as mentioned by Silva et al. (2016), with adaptations. The samples were analyzed using a 10 × magnification. Images were recorded isothermally at 20°C and during heating at 40°C, 60°C, and 80°C at 5°C/min using a Linkam thermal system (Linkam Scientific Instruments Ltd., Redhill, UK) attached to the microscope stage (Valoppi et al. 2023). Images of each sample were observed (100 µm in scale) using Image Pro‐Plus software version 7.0 for Windows.

#### 2.3.3 Color Measurement

Color measurement (L*a*b*) was conducted according to Adrah, Ananey‐Obiri, and Tahergorabi (2021) and the whiteness index (WI) values were calculated as mentioned by Li et al. (2021). The measurements were taken through the glass vial surface, and the parameters were measured at three random surface areas of each sample.

#### 2.3.4 Oil Binding Capacity (OBC)

The OBC of the samples was determined by accelerated stability tests using the method mentioned by Ferdaus et al. (2022). Thus, after weighing 1 g of sample into a 1.5 mL centrifuge tube, the samples were centrifuged for 15 min using a high‐speed centrifuge at 10,000 rpm at 25°C. After centrifugation, the tubes were inverted for 30 min, the discharged oil was absorbed with filter paper, and the oil retention capacity was calculated as shown in Equation (1):

(1) $$\mathrm{OBC}\;\;\left(\%\right)=\left[1-\left(\frac{m_{i}\;-\;m_{f}}{m_{i}}\right)\right]\times 100$$

where *m_i_* (g) is the weight of the sample before centrifugation and *m_f_* (g) is the weight after draining the oil.

#### 2.3.5 Thermal Behavior

The thermal behavior of BOGs and BEGs was analyzed using a differential scanning calorimeter (DSC; TA Instruments, New Castle, DE, USA). Approximately 8–12 mg of each sample was accurately weighed and hermetically sealed in an aluminum pan, with a sealed empty pan as the reference. Before analysis, the samples were equilibrated at 20°C for 1 min to ensure stabilization within the DSC chamber. The samples were then cooled to −10°C to facilitate complete crystallization. After holding at −10°C for 10 min, the samples were subjected to a heating cycle, melting from −10°C to 90°C (BOGs) and from −10°C to 200°C (BEGs) at a controlled rate of 5°C/min under a continuous nitrogen gas flow (50 mL/min). Key thermal parameters, including onset temperature (°C), peak height (W/g), peak temperature (°C), and melting enthalpy (ΔH, J/g), were determined from the melting curves. All measurements were performed in triplicate and on a wet basis.

#### 2.3.6 Rheological Measurements

To evaluate the viscoelastic properties of the samples (24 h after production), dynamic oscillatory tests (strain sweep) were determined using a rheometer (Discovery HR‐10, TA Instruments, New Castle, DE, USA). The upper fixture was a parallel plate (25 mm), and about 4 g of sample was put into the rheometer and trimmed to remove the excess outside of the fixture when the gap was 2050 µm. Initially, the strain sweep test was performed in the strain range 0.1%–100% (at a constant frequency of 1 Hz and a temperature of 25°C) to determine the following parameters: (1) linear viscoelastic range (LVR or γ LVR); (2) viscoelastic behavior in the LVR range; (3) crosspoint of storage modulus (G') and loss modulus (G'') (flow point, G' = G''). Moreover, no specific anti‐slip or evaporation control system was applied during the rheological measurements.

#### 2.3.7 Statistical Analysis

Experiments were conducted in triplicate, and all results are presented as mean ± confidence interval in total sample mass (wet matter). Significance (*p* < 0.05) was assessed by one‐way ANOVA and Tukey test were performed using Statistica 7.0 software (Statsoft Inc., Tulsa, OK, USA).

## 3 Results and Discussion

### 3.1 Macroscopic Appearance

**FIGURE 1:**
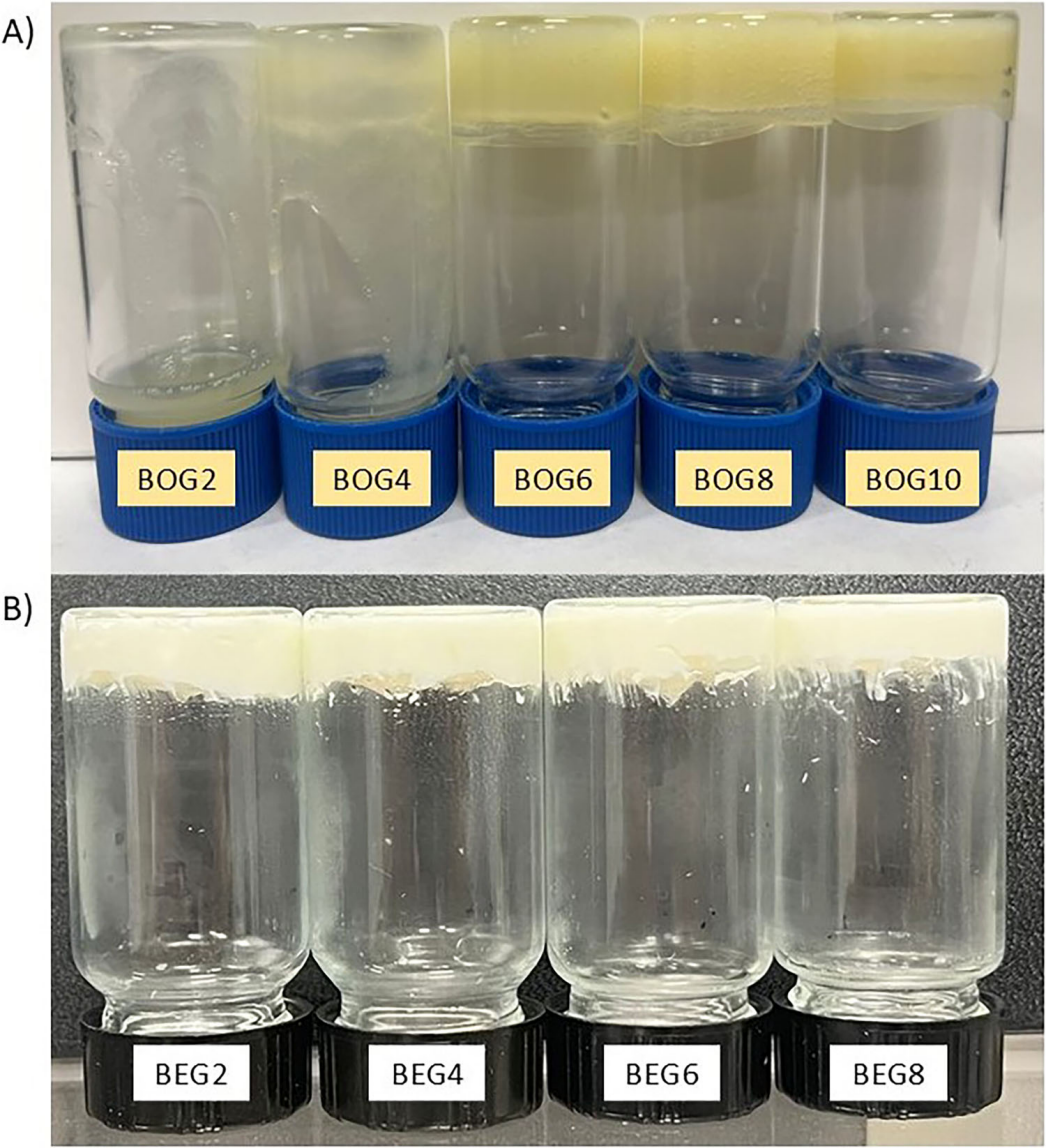
Macroscopic appearance: (A) Brazil nut oleogels (BOG) with carnauba wax at different additions; and (B) Brazil nut emulgel (BEG) at different proportions of gelatin at 25°C.

Figure 1A presents the results of the visual analysis of the BOGs prepared with the CW incorporated into BNO. After the system inversion, a transition from a liquid to a semi‐solid state was observed, starting at a 6% CW (w/w) concentration, indicating the formation of the OG. These results demonstrate the loss of fluidity of the system, which is related to the crystalline phase formed by the CW crystals. After dilution of the gelling agent and reduction of the solution temperature, this phase was formed through the crystallization of CW (nucleation) and self‐assembly (mainly driven by branched methyl groups), creating a crystalline network capable of trapping BNO (Shahamati et al. 2024; Doan et al. 2015).

The high amounts of wax esters (58%–62%) and fatty alcohols (30%–34%) in CW influence its gelation behavior. Thus, because these compounds play key roles in the oil gelation process, CW tends to show a lower gelation temperature (25°C–45°C) and requires a higher concentration to form gels compared with other waxes (Doan et al. 2017, 2015). Additionally, studies indicate that concentrations of 4%–6% CW are necessary to gel oils with elevated unsaturated fatty acid (UFA) levels, attributed to the presence of medium‐ and long‐chain triglycerides, such as oleic and linoleic acids (R. Wang et al. 2024; Buitimea‐Cantúa et al. 2021). The high UFA content in BNO (∼70%), reported previously by our research group (Santos et al. 2025), aligns with findings from previous studies.

On the other hand, BEGs were formed at all concentrations tested, indicating that gelatin was efficient in structuring the oil into semi‐solid systems (Figure 1.B). This result was achieved because gelatin is an amphipathic protein able to adsorb to the oil‐water interface of oil droplets (Lee et al. 2025). According to Y. Lin et al. (2025), gelatin‐based emulsion gels belong to the class of emulsion‐filled gels, in which the continuous phase (triple helices) forms an ordered network structure through gelatin's physical self‐assembly or covalent crosslinking. Meanwhile, the dispersed oil droplets serve as “active fillers”, contributing to improved mechanical properties.

### 3.2 Polarized Light Microscopy (PLM)

**FIGURE 2:**
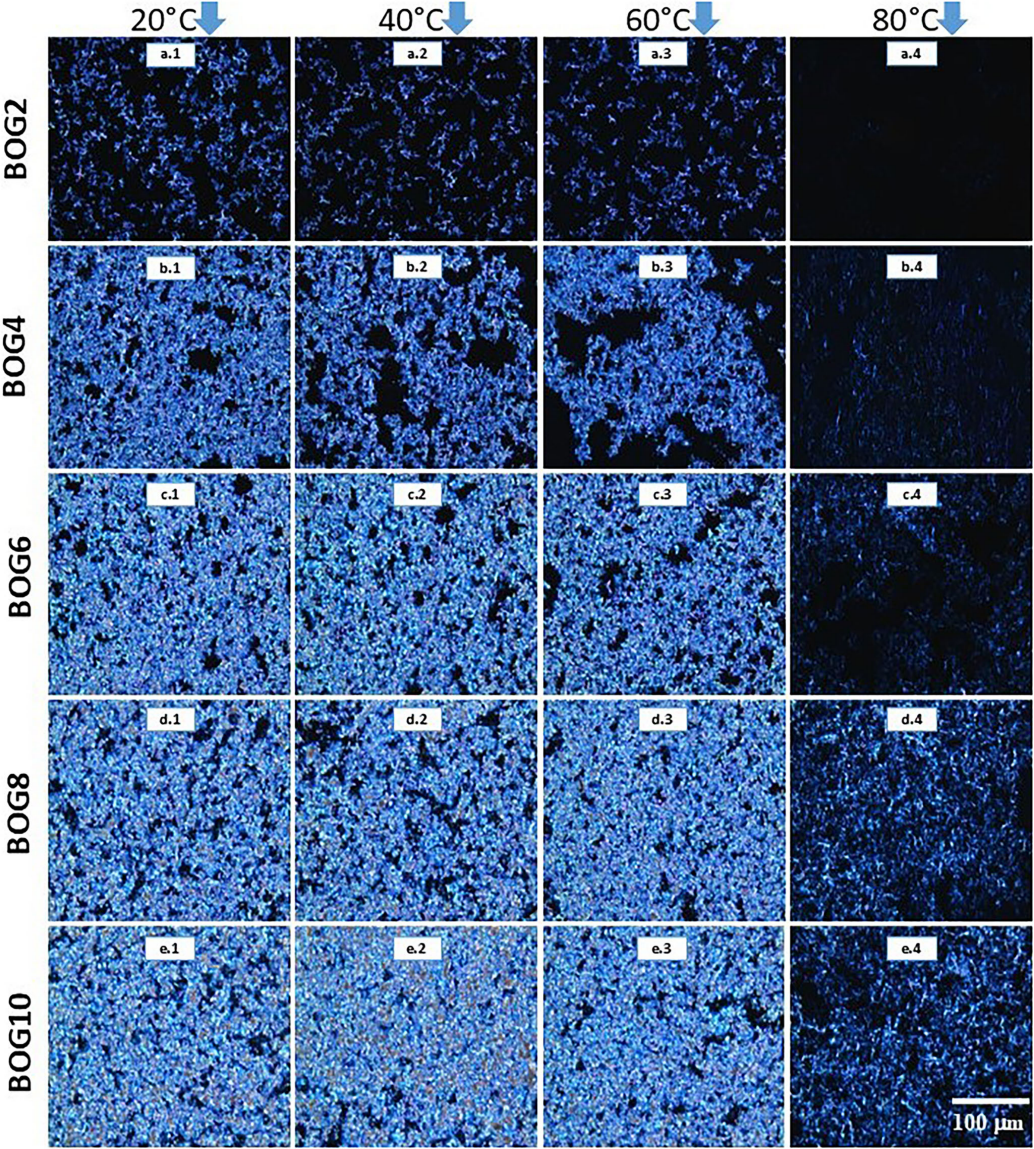
Polarized light microscopic images (a), (b), (c), (d), and (e) of Brazil nut oleogels (BOG) at different proportions of carnauba wax at different temperature (the numbers from left to right indicate the increase in temperature: 1. 20°C; 2. 40°C; 3. 60°C; and 4. 80°C).

PLM analysis of the BOG samples (Figure 2) reveals homogeneous crystals and an increasingly aggregated structure with higher CW concentrations, suggesting the development of a more compact three‐dimensional network driven by physical attractive forces, such as van der Waals interactions (Shahamati et al. 2024). These intermolecular interactions likely arise from aliphatic chains between CW and BNO fatty acids. Notably, the crystals become more pronounced and compact at CW concentrations of 6% or higher, immobilizing the liquid oil within the matrix and maintaining stability up to 60°C (Figure 2c–e). This finding aligns with the macroscopic appearance discussed in Section 3.1.

**FIGURE 3:**
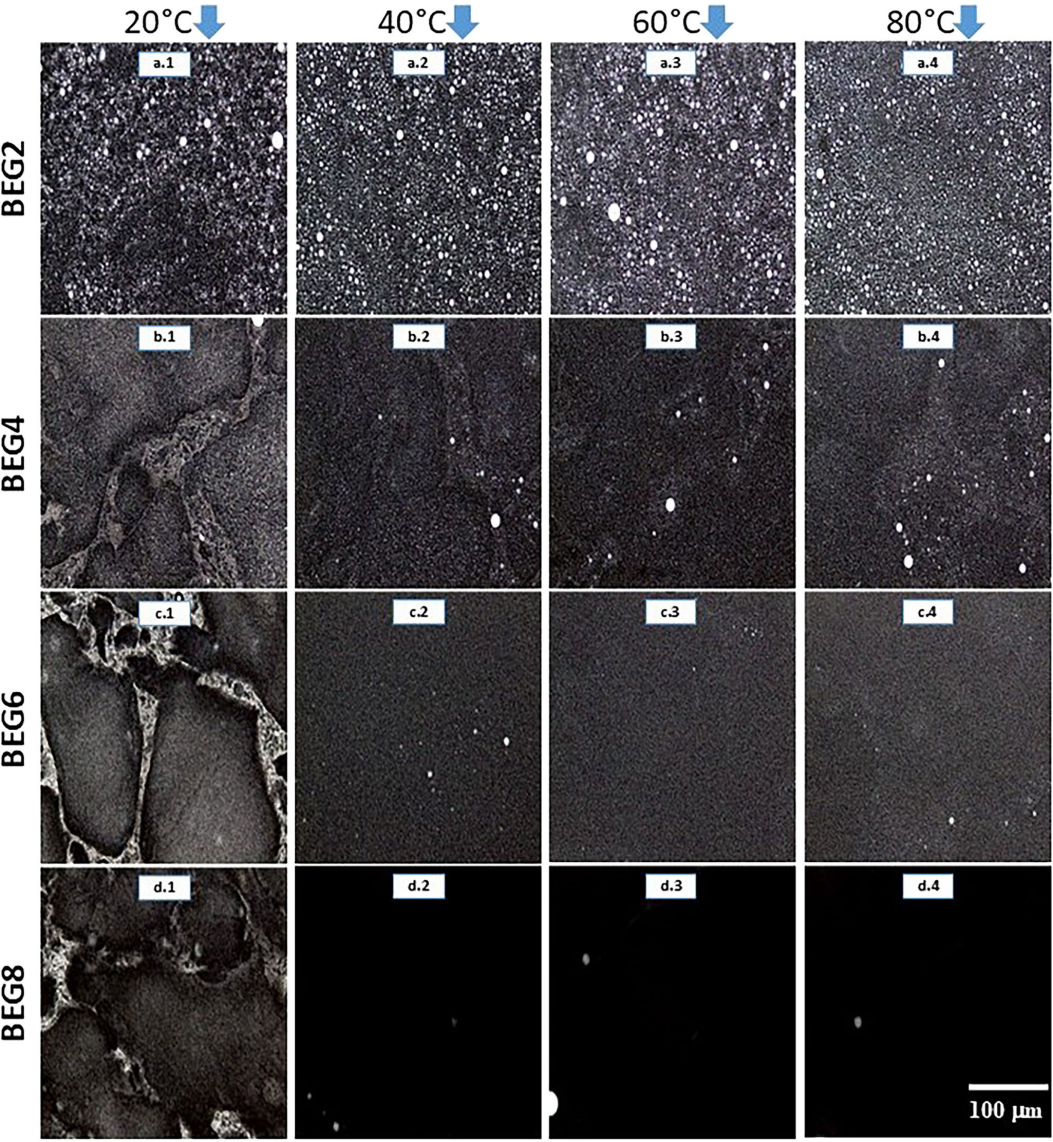
Polarized light microscopic images (a), (b), (c), (d), and (e) of Brazil nut emulgel (BEG) at different proportions of gelatin at different temperature the numbers from left to right indicate the increase in temperature: 1. 20°C; 2. 40°C; 3. 60°C; and 4. 80°C).

Figure 3 illustrates the microstructure of various BEG formulations at different temperatures. The micrographs of the BEG containing 2% gelatin (Figure 3a) show spherical oil droplets dispersed within the hydrogel continuous phase at 20°C. In contrast, micrographs of formulations with gelatin concentrations exceeding 4% (Figure 3c–d) reveal denser, thicker, and more compact EGs. All samples exhibited a structure similar to the emulsion‐filled gels, suggesting oil entrapment within the three‐dimensional network, consistent with the visual analysis findings. In this case, this phenomenon occurs due to the gelation mechanism of gelatin (cold set after heat treatment). Thus, a self‐assembly mechanism of gelatin happens, and helices are established (below 30°C), which leads to aggregation and gelation (Farjami and Madadlou 2019; D. Lin et al. 2020; Nicolai 2019).

Moreover, preliminary tests were conducted using stabilizers such as Tween 20, Tween 40, soy lecithin, glycerol, and *Yucca schidigera* extract in EGs formulated with conventional oils. Notably, Tween 20 demonstrated effective gelatin stabilization by binding to gelatin's peptide bonds and protonated amino groups through hydrogen bonding (Tan et al. 2023). Additionally, all samples lost their structure after reaching 40°C due to the protein denaturation (heat treatment above 40°C, the polypeptide chains lose its rigid tertiary structure and become more mobile, increasing the solubility of gelatin) (Nicolai 2019).

### 3.3 Color Measurement

Table 1 presents the measured color parameters of BOG and BEG, which are important parameters for quality control, indicators of stability, and indicators of consumer acceptance. The luminosity values (L*) ranged from 34.87 ± 1.67 to 50.54 ± 2.00 and from 59.19 ± 4.16 to 69.04 ± 2.53 for BOG and BEG, respectively. Regarding the WI, it ranged from 34.87 ± 1.67 (BOG2) to 68.98 ± 2.54 (BEG8). For both of them, the values presented a significant difference with an increase in the concentration of CW and gelatin (*p* < 0.05). With the increase in the concentration of structuring agents, there is a greater formation of three‐dimensional networks that intensifies the physical effects. These results are aligned with visual and PLM results.

**TABLE 1:** Color parameters result for Brazil nut oleogels (BOG) and emulsion gels (BEG).

Samples	Color			
	$L^{*}$	$a^{*}$	$b^{*}$	Whiteness index (WI)
BOG2	34.87 ± 1.67^g^	−0.87 ± 0.12^a^	0.04 ± 0.21^fg^	34.87 ± 1.67^g^
BOG4	39.35 ± 2.55^f^	−1.57 ± 0.22^b^	0.57 ± 0.35^de^	39.33 ± 2.54^f^
BOG6	45.10 ± 1.68^e^	−2.28 ± 0.10^c^	2.15 ± 0.58^c^	45.01 ± 1.67^e^
BOG8	46.14 ± 5.18^e^	−2.81 ± 0.54^d^	3.89 ± 0.53^b^	45.92 ± 5.11^e^
BOG10	50.54 ± 2.00^d^	−3.14 ± 0.40^e^	4.65 ± 0.47^a^	50.23 ± 1.92^d^
BEG2	59.19 ± 4.16^c^	−1.03 ± 0.04^a^	−0.36 ± 0.29^g^	59.18 ± 4.16^c^
BEG4	61.62 ± 4.78^bc^	−1.40 ± 0.22^b^	0.11 ± 0.29^f^	61.59 ± 4.78^bc^
BEG6	64.56 ± 3.97^b^	−1.39 ± 0.10^b^	0.27 ± 0.06^ef^	64.53 ± 3.96^b^
BEG8	69.04 ± 2.53^a^	−1.56 ± 0.17^b^	0.98 ± 0.27^d^	68.98 ± 2.54^a^

* Values are mean ± confidence interval at a 95% level of significance;.

** Different letters superscripted within the same column represent significant differences (*p* < 0.05).

*** L* = color intensity, L* = 100 for lightness, and L* = 0 for darkness; +a* = increasing red, −a* = increasing green; +b* = increasing yellow, −b* = increasing blue.

For a* that represents the redness, the increase in the concentration of CW and gelatin impacted their reduction, ranging from −3.14 ± 0.40 to −0.87 ± 0.12 and −1.56 ± 0.17 to −1.03 ± 0.04. The yellowing (b*) of BOG10 was higher among the BOG samples compared to the BEG samples. The results indicate that the natural yellowish color of CW (pigments) impacted the OG color. CW contains compounds (mainly chlorophyll and carotenoids), which cause green and yellow‐orange colorations (Buitimea‐Cantúa et al. 2021; Roufegarinejad et al. 2024).

### 3.4 Oil Binding Capacity (OBC)

**FIGURE 4:**
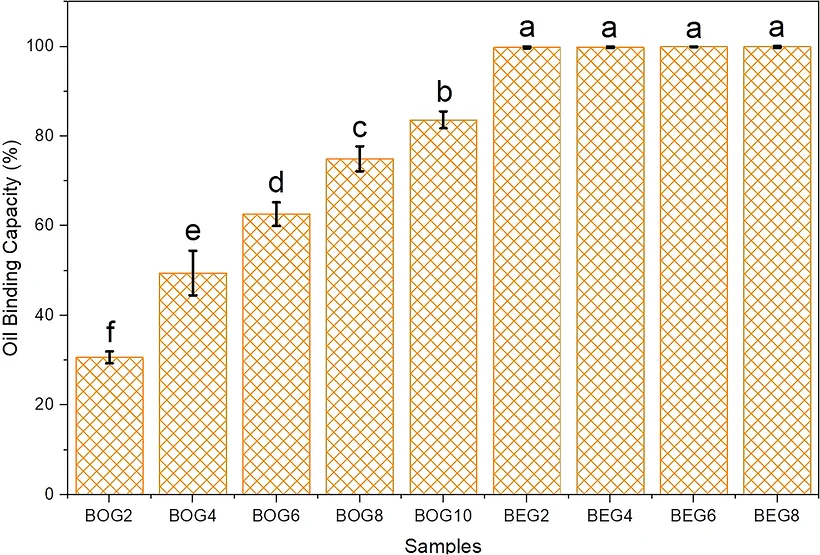
OBC of oleogels (BOG) and emulsion gels (BEG). Different lowercase letters represent different results (*p* < 0.05) according to Tukey's multiple comparison test for the analyzed samples.

OBC is crucial in the development of OGs and EGs, as it allows the assessment of structural stability, texture, and functionality (Pakseresht et al. 2023; Shahamati et al. 2024). High OBC ensures effective oil retention, minimizing oil leakage and increasing gel strength and consistency (Chen et al. 2023). The OBC values for BOG increased significantly (*p* < 0.05) with increasing CW concentrations. For example, the OBC of BOG2 and BOG10 was found to be 30.63% ± 1.34% and 83.65% ± 1.90%, respectively (Fig. 4). This result is consistent with the visual observations and microscopy images, which demonstrate that higher CW concentrations enhance the oleogel network's ability due to the high content of wax esters and hydrocarbons (Qu et al. 2024). Besides, according to Doan et al. (2017), with more CW mass, the hydrogen bonding among long‐chain free fatty alcohols in CW is more pronounced (such as C_30_, C_32_, and C_34_). These findings also align with similar results reported by Thakur et al. (2022) and Shi et al. (2022), where a high wax content enhances the OG's ability to retain liquid oil, resulting in a higher OBC.

In contrast, the results for BEG were significantly superior (*p* < 0.05) to those for BOGs. All BEG samples exhibited an OBC of approximately 100% with no significant differences observed (*p* > 0.05). This behavior can be associated with the presence of high fatty alcohol content and uneven distribution of dendritic and aggregate‐like crystals in CW (BOG), which form unstable networks and limit their ability to retain large amounts of oil in comparison to the gelatin (BEG) (Z. Wang et al. 2023, 2024; Qu et al. 2024). Moreover, the amphiphilic protein structure and electrostatic interactions of gelatin, combined with the presence of Tween 20 in BEG, enable the proteins and surfactants to migrate toward the lipid interface, reducing surface tension and preventing droplet coalescence (Taktak et al. 2021; Wu et al. 2024). In addition, the gelatin's active surface contains charged and amphiphilic groups (e.g., NH_2_‐ and OH‐), which facilitate hydrogen bond formation at the oil‐water interface, enhancing emulsion stability, resulting in a more stable structure compared to BOGs (Y. Wang et al. 2025; Ahmad et al. 2024). According to Zeng et al. (2023), gelatin's sol‐gel transition—in which single chains associate into thermally reversible triple helices stabilized by hydrophobic interactions, hydrogen bonds, and van der Waals forces—builds a continuous viscoelastic network that entraps oil droplets and increases stability of BEGs.

### 3.5 Thermal Behavior (DSC)

In Table 2, BNO, BOG, and BEG thermal behaviors are presented. The BNO peak is regarding the concentration of UFAs, mainly oleic fatty acid (Santos et al. 2025). The BOG thermal profiles presented a melting peak, where the endothermic peak ($TC_{\mathrm{onset}}$ from 58.64°C ± 1.83°C to 65.55°C ± 0.60°C) is regarding the composition of CW and presented a high melting behavior compared with other waxes (Ferdaus et al. 2022) due to the high concentrations of wax ester and free fatty alcohol, as aforementioned (Doan et al. 2015, 2017). For the BEG melting profile, one peak was observed with $TC_{\mathrm{onset}}$ from 89.68°C ± 1.05°C to 103.74°C ± 1.33°C. As mentioned by Corredor‐Chaparro et al. (2022), endothermic behavior between 85°C and 120°C is due to the presence of gelatin which is associated with the helix‐coil transition of gelatin (Obas et al. 2024) and can be also attributed to polyproline residues (Mukherjee and Rosolen 2013). Moreover, considering ∆H as enthalpy required for melting (Atik et al. 2022), BEG showed the highest data compared with BOG. As mentioned by T. Huang et al. (2018), the thermoreversible behavior of gelatin is associated with the difference in energy required for the association and dissociation of the junction zones between its filaments. This process directly influences the gelation and melting points, with higher gelation points tending to result in greater thermal stability. According to Qiao et al. (2018), the length of the triple helices in gelatin is closely linked to the thermal stability and the melting enthalpy is proportional to the triple helix content. Besides, comparing the melting profiles of BEG, BOG, and commercial fats such as butter and shortening (15°C–45°C) (Nutter et al. 2023), the structured systems exhibited higher melting onsets (58.6°C–103.7°C), indicating that their suitability is dependent on the specific food application.

**TABLE 2:** Thermal stability differential scanning calorimetry (DSC) results for Brazil nut oleogels (BOG) and emulsion gels (BEG).

Samples	Peak				
	$TC_{\mathrm{onset}}$	$TC_{\mathrm{endset}}$	$H_{E}$	$T_{p}$	ΔH
	(°C)	(°C)	(W/g)	(°C)	(J/g)
BNO	−8.50 ± 1.89^g^	2.57 ± 1.22^d^	−0.15 ± 0.01^a^	−0.77 ± 0.86^f^	14.38 ± 0.86^f^
BOG2	58.64 ± 1.83^f^	78.43 ± 1.14^c^	−0.01 ± 0.01^a^	70.43 ± 2.98^e^	1.51 ± 0.20^h^
BOG4	62.38 ± 1.34^e^	80.63 ± 5.55^c^	−0.02 ± 0.02^a^	71.61 ± 0.35^de^	2.70 ± 0.50^gh^
BOG6	63.67 ± 1.70^de^	77.25 ± 9.99^c^	−0.04 ± 0.03^a^	73.36 ± 0.40^de^	5.86 ± 0.74^g^
BOG8	65.55 ± 0.60^d^	79.27 ± 0.88^c^	−0.08 ± 0.01^a^	73.98 ± 1.91^de^	13.13 ± 2.01^f^
BOG10	65.48 ± 4.12^d^	80.08 ± 0.53^c^	−0.11 ± 0.03^a^	75.56 ± 0.96^d^	17.74 ± 5.86^e^
BEG2	89.68 ± 1.05^c^	123.75 ± 2.42^b^	−11.446 ± 0.60^c^	96.95 ± 1.63^c^	726.77 ± 3.59^a^
BEG4	97.53 ± 6.06^b^	124.41 ± 0.18^b^	−5.070 ± 0.83^b^	103.81 ± 4.65^b^	706.41 ± 1.02^b^
BEG6	98.37 ± 0.53^b^	128.89 ± 1.23^b^	−7.359 ± 8.42^b^	106.79 ± 8.29^b^	533.30 ± 4.24^c^
BEG8	103.74 ± 1.33^a^	142.71 ± 9.37^a^	−6.418 ± 12.87^b^	133.72 ± 12.97^a^	491.48 ± 6.75^d^

* Values are mean ± confidence interval at a 95% level of significance.

** Different letters superscripted within the same column represent significant differences (*p* < 0.05).

$T_{o}$—Onset temperature; $H_{E}$—Peak height; $T_{p}$—Peak temperature; and ∆H—Enthalpy.

Although the higher melting temperatures indicate improved thermal stability, they may also limit the applicability of these structured systems in products requiring rapid melting at oral temperature. Depending on the food application, increased melting temperatures may reduce spreadability, delay flavor release, and produce a waxy mouthfeel, thereby influencing consumer perception (Espert et al. 2021; Guichard, Galindo‐Cuspinera, and Feron 2018). Conversely, this enhanced thermal stability may be advantageous for products requiring structural integrity during processing or storage (Aliasl khiabani et al. 2020). Therefore, the suitability of these systems should be evaluated according to the specific technological requirements of the intended food application, and future studies should include sensory and application‐based assessments.

### 3.6 Rheological Measurements

**FIGURE 5:**
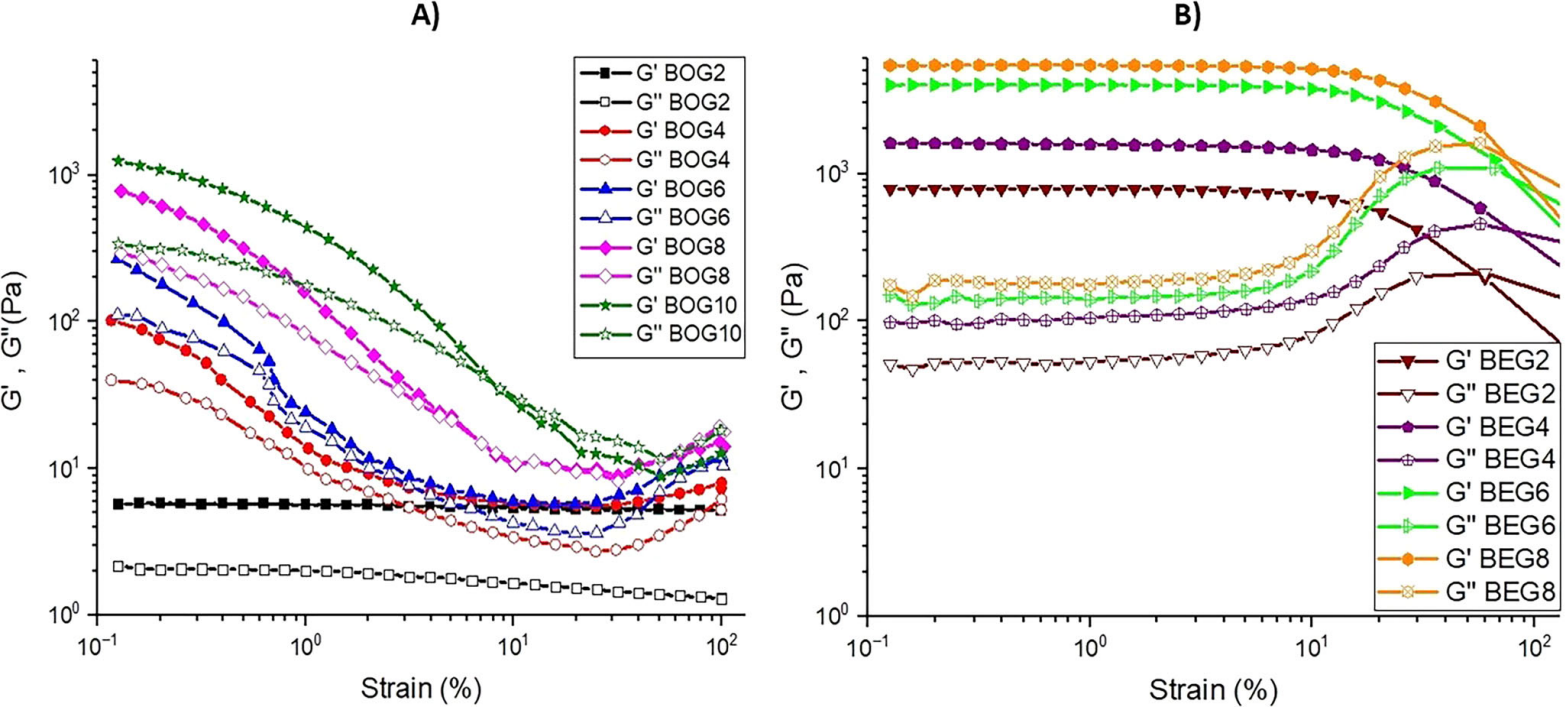
Rheological behaviors of emulsion gels (BEG) and oleogels (BOG). Plots show the log‐log plot of the storage modulus (G′) and loss modulus (G″) as a function of applied strain for (A) BOG and (B) BEG.

In the development of gels, rheological characterization is critical and provides comprehensive insights into structural integrity and functional performance. By assessing key parameters, rheological analysis elucidates the textural properties, spreadability, and mechanical stability of these systems. This information is crucial for optimizing formulations to replicate the sensory and functional properties of conventional fats while ensuring product stability throughout processing, storage, and consumption. Figure 5 presents the relation of the elastic component (G') and the viscous component (G'') on the oscillation strain applied to the BOGs and BEGs. It is possible to notice that for all BOG samples present, the G' prevails over the G'' at small applied shear, reflecting that the BOGs had a predominantly elastic rather than viscous nature, characteristic of solid/semi‐solid gels (Figure 5A). On the other hand, it does not correlate with the linear response region (LVR). With increasing strain, the G' value starts to decrease, indicating structural deformation or breakdown, which can be related to the formation of a fat‐like crystal network, as mentioned in the PLM topic (Patel et al. 2013). The crossover point (G′ = G″), which indicates the transition from solid‐like to liquid‐like behavior, varied among the samples. BOG6 exhibited the highest crossover stress (11.7 Pa) and strain (99%), suggesting a stronger and more deformation‐resistant network. In contrast, BOG8 and BOG10 showed lower crossover values (approximately 10.8 ± 1.6 Pa and 7.0% ± 1.5% strain, respectively), indicating weaker structures that yield under lower deformation.

In contrast, BEGs exhibited typical gel‐like behavior (G′ > G″; Figure 5B), with both moduli remaining nearly constant within the linear viscoelastic region (LVR) up to a critical strain of approximately 12%. Beyond this point, the structure began to break down, as evidenced by a sharp decrease in G′. Simultaneously, G″ showed an initial increase followed by a pronounced decline, indicating energy dissipation during network disruption. This response is characteristic of type III behavior (weak strain overshoot) (Zhao et al. 2022), suggesting partial structural rearrangement prior to complete breakdown. Additionally, BEGs displayed a higher crossover point at approximately 100%, signifying the transition from semi‐solid‐like to liquid‐like behavior. This transition likely results from the disruption of intermolecular polymer bonds within the network structure under large deformations, leading to network yielding (Zhao et al. 2022). These results indicate that the BEGs developed in this study could effectively withstand higher stress and better maintain their original gel structure compared to BOGs. However, future studies should include frequency sweep analyses to provide a more comprehensive evaluation of the rheological properties. Moreover, no anti‐slip fixture or evaporation control system was used during rheological measurements. Although all samples were analyzed under identical conditions and the tests were completed within a relatively short time, the absence of these controls may have influenced the measured rheological properties and should be considered a methodological limitation.

In comparison to the development of EG based on gelatin and European eel oil, it was not possible to observe a stable structure (without LVR), but it presented a crossover point of approximately 650% for the sample at 20°C (Taktak et al. 2021). Recent studies have demonstrated that the physicochemical and rheological properties of these systems are strongly dependent on the type and concentration of gelling agents, as well as the interactions within the network, which ultimately influence their performance in food applications (Younis et al. 2026; Alamer et al. 2025).

## 4 Conclusion

In this study, OG and EG were successfully produced using BNO structured with CW and gelatin. Concentrations above 6% CW were required to effectively structure BNO, while only 2% gelatin was sufficient to promote the liquid‐to‐semi‐solid transition. PLM and OBC results indicated that CW‐driven crystallization governed OG structuring, whereas EG presented a more stable three‐dimensional network, maintaining oil‐binding values close to 100% across all formulations. Rheological analysis showed that only BOG6, BOG8, and BOG10 exhibited G' > G'', while all EG samples demonstrated semi‐solid behavior with greater stability than OG. In particular, BEG2 exhibited promising potential due to its ability to form a semi‐solid structure at a low gelling‐agent concentration while maintaining high oil‐binding capacity and favorable rheological properties. Further application studies are required to evaluate its performance as a potential fat model, as its suitability will depend on the specific formulation and processing requirements of each application. Additionally, future studies such as thermodynamic properties and evaluating storage stability (including oil leakage and oxidative stability) are recommended to further elucidate the thermal and structural stability of emulsion gel systems, particularly at moisture contents around 25%. Overall, this work reinforces the technological and socioeconomic potential of Amazon oils for developing healthier structured‐fat alternatives.

## Author Contributions

**Rogério Willian Silva dos Santos**: conceptualization, formal analysis, data curation, writing – original draft, writing – review and editing. **Md. Jannatul Ferdaus**: formal analysis, data curation, writing – original draft, writing – review and editing. **Marcos Rogério Mafra**: resources, project administration, supervision, validation, visualization, writing – review and editing. **Roberta Claro da Silva**: resources, project administration, supervision, validation, visualization, writing – review and editing. **Tirzhá Lins Porto Dantas**: funding acquisition, resources, project administration, supervision, validation, visualization, writing – review and editing.

## Funding

This study was financed by the Coordination of Improvement of Higher Education Personnel—CAPES (Finance Code 001), Grant/Award Numbers: 88887.837383/2023‐00; and 88881.934242/2024‐01.

## Conflicts of Interest

The authors declare no conflicts of interest.
